# An Efficient Extraction, Characterization and Antioxidant Study of Polysaccharides from *Peucedani Decursivi* Radix

**DOI:** 10.3390/plants14142188

**Published:** 2025-07-15

**Authors:** Qian Li, Zeyu Li, Chaogui Hu, Chenyue Wang, Feng Yang, Xiaoqin Ding

**Affiliations:** State Key Laboratory of Aridland Crop Science, College of Agronomy, Gansu Agricultural University, Lanzhou 730070, China; lizy092023@163.com (Z.L.); hcg1949hcg@outlook.com (C.H.); wcy04042023@163.com (C.W.); 16693768162@163.com (F.Y.); dingxq1026@126.com (X.D.)

**Keywords:** *Peucedani Decursivi* Radix, polysaccharides, ultrasonic-assisted extraction, natural deep eutectic solvents, antioxidant activity

## Abstract

Hot water extraction (HE), enzyme-assisted hot water extraction (EAHE), ultrasonic-assisted extraction with NADES (UAE-NADES) and ultrasonic-assisted extraction with NADES and enzyme pretreatment (UAE-NADES-E) were employed to extract polysaccharides from *Peucedani Decursivi* Radix (PDR) and their structures were characterized for the first time. UAE-NADES-E was found to be the most effective extraction method, and the extraction process was optimized by Box–Behnken design (BBD)-response surface methodology (RSM) experiments. The optimal extraction process was determined by using a NADES system with a molar ratio of betaine to 1,3-butanediol of 1:3, a water content of 30%, a liquid/solid ratio of 40:1 mL/g, an ultrasound time of 30 min, an ultrasound temperature of 45 °C and an alcohol precipitation time of 6 h; the polysaccharide extraction yield reached 19.93%. Further, the structures of polysaccharides from PDR extracted by the above four methods were characterized by FT-IR, SEM, gel and anion-exchange chromatography. Eight monosaccharides were detected in the PDR polysaccharides extracted by the four methods. The PDR polysaccharides extracted by the UAE-NADES-E method had lower molecular weights compared with those extracted by the other methods. Moreover, the PDR polysaccharides exhibited obvious antioxidant activity, as revealed by DPPH, ABTS+ and hydroxyl radical scavenging experiments, meaning they have the potential to be developed as natural antioxidants.

## 1. Introduction

The umbelliferous plant *Peucedanum decurvivum* (Miq.) Maxim is a perennial herb that grows in meadows, beside hillside forest streams and in hillside sparse forests and is mainly distributed in Anhui, Zhejiang, Jiangxi, Jiangsu, Hunan, Guizhou and other provinces of China. *Peucedani Decursivi* Radix (PDR) is the dried root of *Peucedanum decurvivum* (Miq.) Maxim, and has been used as a traditional Chinese medicine with the effect of evacuating wind-heat, reducing gas and eliminating phlegm [1,2]. The present research shows that there are a variety of chemical components, including coumarins, volatile oils, flavonoids, fatty acids and other components in PDR [3,4].

As is known, high concentrations of polysaccharides are often found in plants. Polysaccharides are the structural substances of plant cells and the main source of cell energy. Recent studies have shown that polysaccharides have a range of effects including antioxidant, anti-tumor, immune regulation, antimicrobial, anticoagulant, anti-diabetes, antiviral and hypoglycemic activities [5,6,7]. On the other hand, Alzheimer’s disease, diabetes and cardiovascular diseases are confirmed to be related to in vivo oxidative stress [8,9]. It is of great significance to explore the structure and antioxidant properties of polysaccharides. However, much effort has been focused on the coumarins; the polysaccharides from PDR are rarely explored, so their structures and biological activities are unknown. Efficient extraction and structural characterization is essential to further study and utilize the polysaccharides from PDR.

The traditional method for extracting polysaccharides from plants is hot water reflux, which is a time-consuming and high-temperature method that results in a low yield. To achieve high yields of polysaccharides, various pretreatment methods to separate biomass components have been developed, including physical, chemical, physical–chemical and biological approaches [10]. Ultrasonic-assisted extraction (UAE) is one of the most important physical pretreatment methods; it has the advantages of a shorter extraction time and high extraction efficiency [11]. Enzyme-assisted extraction is one of the biological approaches; this method can degrade and disrupt the cell wall structures of the plant and significantly improve the polysaccharide extraction rate. Furthermore, significant progress has been made in the extraction of the active components from plants by using enzyme pretreatment combined with ultrasonic-assisted extraction technology [12]. The structure of the plant cell wall can be destroyed and hydrolyzed through enzyme and ultrasonic technology, so that the effective components in the plant can be fully dissolved and the extraction rate of effective components is improved.

In recent years, deep eutectic solvents (DESs) have emerged in new techniques of biomass fractionation and can improve the extraction efficiency [13,14,15]. Abbott et al. proposed, for the first time, that DESs [16] are stable solvents formed by combining hydrogen bond acceptors (such as quaternary ammonium salts) with hydrogen bond donors (such as alcohols, carboxylic acids, amides, etc.) on the basis of hydrogen bond bridging. Natural deep eutectic solvents (NADESs), formed by natural primary metabolites including organic acids, sugars, amino acids, urea, choline derivatives and alkaloids, serve as a green alternative to DESs [17,18]. They are non-toxic, inexpensive, simple to prepare and biodegradable green solvents. In previous studies, using choline chloride as the hydrogen bond acceptor has garnered great attention. In particular, betaine is extracted from sugar beets, which is much cheaper and environmentally friendly and could act as an alternative to choline chloride [19,20,21,22]. In recent years, ultrasonic-assisted DES and NADES extraction has been widely used for extracting and separating bioactive components from plants [23]. In particular, DESs and NADESs have emerged as promising solvents for the extraction of polysaccharides from various sources, showing enhanced efficiency in terms of polysaccharide extraction yield compared to conventional solvents. Combining DES and NADES extraction with microwave, ultrasound and enzymatic degradation methods could achieve a higher yield of products. DESs and NADESs also have the potential to modify polysaccharide characteristics and enhance their biological activities through changing their structural features [24,25].

In this work, for the first time, hot water extraction (HE), enzyme-assisted hot water extraction (EAHE), ultrasonic-assisted extraction with NADESs (UAE-NADES) and ultrasonic-assisted extraction with NADESs and enzyme pretreatment (UAE-NADES-E) were employed to extract polysaccharides from *Peucedani Decursivi* Radix (PDR). NADESs with choline chloride and betaine as the hydrogen bond acceptors were prepared. An efficient and environmentally friendly method was developed to extract the polysaccharides from PDR using NADESs combined with ultrasound-assisted enzyme pretreatment. The extraction yield of polysaccharides was measured by the phenol–sulfuric acid method. The optimal extraction process for polysaccharides from PDR was obtained based on a single factor and response surface methodology experiments. Further, the effect of extraction methods on the chemical structure of polysaccharides from PDR was characterized by Fourier-transform infrared (FT-IR) spectroscopy, gel and anion-exchange chromatography. The surface morphology of polysaccharides was observed by scanning electron microscopy (SEM) analysis. The antioxidant activity of polysaccharides was studied by scavenging of hydroxyl (OH) radicals, 2,2′-Azino-bis (3-ethylbenzothiazoline-6-sulfonic acid) (ABTS) radicals and 2,2-Diphenyl-1-picrylhydrazyl (DPPH) radicals, which have been widely used to evaluate the antioxidant activity.

## 2. Results and Discussion

### 2.1. Screening of the Best NADESs for the Extraction of Polysaccharides from PDR

**Figure 1:**
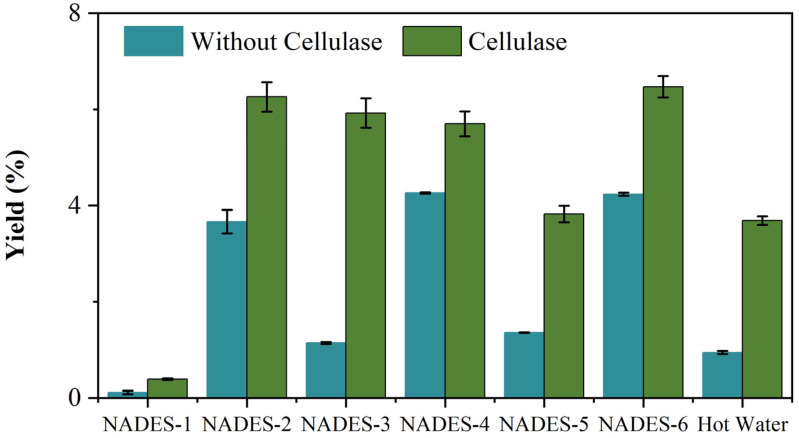
Extraction yields of polysaccharides from PDR by NADESs and hot water with and without enzyme pretreatment.

The results showed that different solvents had an influence on the extraction yield of PDR polysaccharides. To screen the advantages of NADESs in extracting PDR polysaccharides, the extraction yields of the hot water extraction method and different NADESs were compared. In addition, the extraction processes with and without cellulase pretreatment were carried out. The statistical analysis results are shown in Figure 1. The yield of water extraction method for extracting polysaccharides from PDR was only 3.77%, while the UAE-NADES method has a yield of 6.67%. Compared with the hot water extraction method, the extraction yield by NADES-2, NADES-4 and NADES-6 were significantly higher. The water extraction method requires a longer extraction time, whereas the extraction time is significantly reduced with the assistance of ultrasound, addressing the disadvantages of the time-consuming and laborious water extraction method and achieving a higher extraction yield. NADESs are composed of hydrogen bond donors and acceptors, forming a strong hydrogen bond network structure with high polarity and permeability, which could effectively dissolve components such as cellulose, hemicellulose and pectin in plant cell walls, disrupting the cell wall structure and releasing intracellular polysaccharides. NADESs could also form soluble complexes with polysaccharides through van der Waals forces or electrostatic interactions, promoting the dispersion and stability of polysaccharides in solvents, thereby enhancing the extraction efficiency.

Further, the extraction yield of NADES-6 with the cellulose pretreatment is significantly higher than that of other combinations, and the extraction conditions were further optimized using single factor and BBD-RSM experiments.

### 2.2. Single Factor Experimental Optimization Results on the Yields of PDR Polysaccharides

#### 2.2.1. The Effect of Liquid-to-Material Ratio on the Yields of PDR Polysaccharides

**Figure 2:**
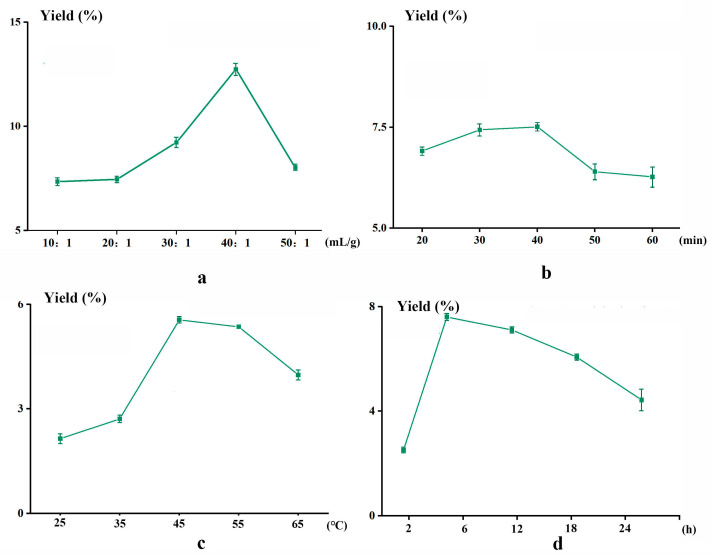
The effect of liquid-to-material ratio (**a**), ultrasonic time (**b**), ultrasonic temperature (**c**) and alcohol precipitation time (**d**) on extraction yields of polysaccharides from PDR.

As can be seen from Figure 2a, the polysaccharide extraction yields increase initially and then decrease as the liquid-to-material ratio ranges from 10:1 mL/g to 40:1 mL/g, reaching a maximum value (12.80 ± 0.05) % at a ratio of 40:1 mL/g. Within a certain range, as the liquid-to-material ratio increases, the reaction area between the solvent and the sample increases, and under the auxiliary effect of ultrasound, the polysaccharides continue to dissolve, thus leading to an increase in polysaccharide extraction yields. However, as the proportion of the NADESs further increases, the polarity of the DES system decreases, which is primarily caused by the dilution effect resulting from excessive solvent volume, reduced solute concentration and accelerated molecular diffusion-driven mass transfer [26]. Consequently, a liquid-to-material ratio of 40:1 mL/g is recommended based on systematic analysis.

#### 2.2.2. The Effect of Ultrasonic Time on the Yields of PDR Polysaccharides

Ultrasonic time significantly influences the extraction yield of polysaccharides. As shown in Figure 2b, the polysaccharide extraction yields increase with prolonged sonication from 20 to 30 min. When the ultrasonic time exceeds 30 min, the extraction yield begins to decline. This decline may be due to the hydrolysis of some polysaccharides under the combined action of high temperature and long extraction time. Therefore, 30 min is identified as the optimal extraction time, achieving the highest extraction rate of (7.61 ± 0.15)% under these conditions.

#### 2.2.3. The Effect of Ultrasonic Temperature on the Yields of PDR Polysaccharides

Figure 2c indicates that the maximum extraction yield (5.53 ± 0.10)% of PDR polysaccharides is achieved at 45 °C. Elevated temperatures accelerate the reaction rate, which is conducive to the dissolution of polysaccharides. Conversely, a further temperature rise may cause damage to the structure of the polysaccharides, leading to a decrease in extraction yield. Therefore, 45 °C was determined to the optimum extraction temperature for polysaccharides in this study.

#### 2.2.4. The Effect of Alcohol Precipitation Time on the Extraction Yields of PDR Polysaccharides

Figure 2d illustrates the effect of alcohol precipitation times on the extraction yields of polysaccharides. The polysaccharide extraction yield increases from 2 to 6 h but decreases beyond this point. As the alcohol precipitation time increases, other macromolecules such as proteins and starch interact and crosslink, forming larger particles and accelerating the sedimentation rate. However, the longer precipitation time may cause the precipitated polysaccharides to decompose into monosaccharides or other substances [27], reducing the yield and recovery rate of polysaccharides. Therefore, 6 h was chosen as the optimal alcohol precipitation time, where the extraction yield reaches (7.75 ± 0.16)%.

### 2.3. BBD-RSM Optimization Results on the Yields of PDR Polysaccharides

The BBD methodology was further employed to optimize the UAE-NADES-E extracting process based on the single-factor optimizing results. The key factors including liquid-to-solid ratio, ultrasonic time, ultrasonic temperature and alcohol precipitation time were studied for their impact on the extraction yields of polysaccharides (Table 1).

The optimal combination of these factors and the experiments were determined based on a BBD-RSM experiment (Table 2). A regression equation was derived for the extraction yields of polysaccharides as a function of the four variables by using Design-Expert 13 software to analyze the data. Y = 7.36 + 2.56 A + 0.068 B + 1.25 C + 0.13 D − 2.56 AB + 0.10 AC + 2.31 AD − 3.52 BC + 0.88 BD −0.3.17 CD − 1.87 A2 +3.22 B2 − 0.69 C2 + 0.90 D2.

The variance analysis of the response surface data obtained from the regression model is shown in Table S1 (Supplementary Materials). The coefficient R2 = 0.9905, indicates the experimental model can be used for prediction within the control variable range. The significance of each coefficient has been quantified by the *p*-values test, the smaller the *p*-value, the more significant the corresponding coefficient. Table S1 (Supplementary Materials) demonstrates that the first-order terms A and C and the second-order terms A2, B2, C2 and D2 are extremely significant (*p* < 0.01). In addition, according to the F value, the factors affecting the polysaccharide extraction yield follow this significant order: A > C > D > B, which means that the liquid/solid ratio is the most influential factor, followed by ultrasonic temperature, alcohol precipitation time and ultrasonic time.

**Figure 3:**
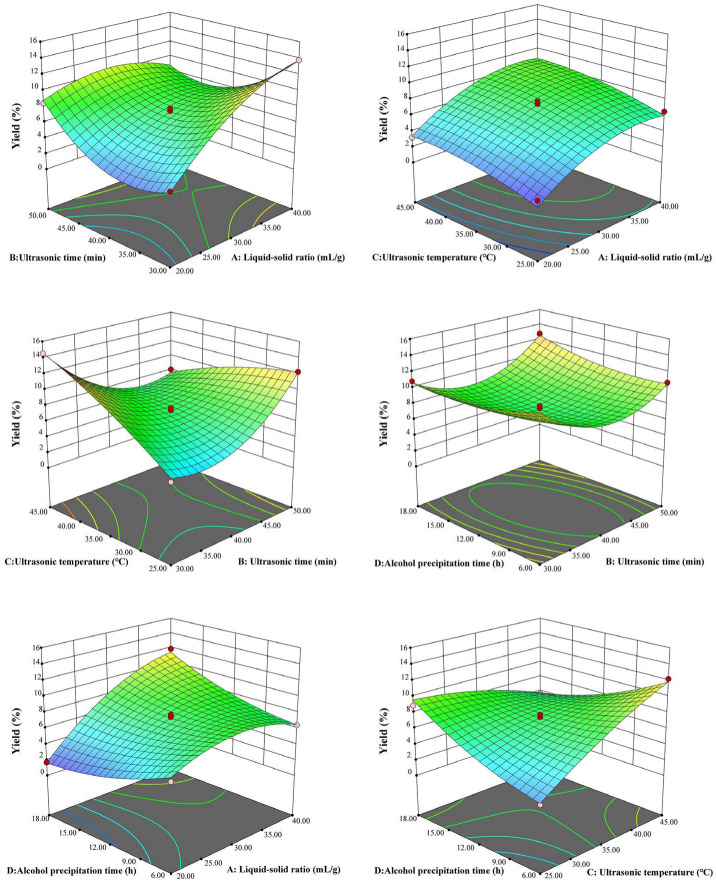
The 3D response surface diagram of the interaction between two independent variables.

Figure 3 displays the three-dimensional surface diagrams created by the Design Expert 13 software, visualizing the interaction between two factors. The steeper the gradient change in the three-dimensional surface, the greater the density of the contours, the more ellipsoidal or saddle-like, suggesting a much stronger interaction between the two factors. Among them, the contours of AB, AD, BC and CD are dense, suggesting that the interaction is significant.

### 2.4. Verification of Prediction Model 

The best variable conditions were determined using Design Expert 13 software. The optimized process conditions are as follows: liquid-to-solid ratio of 37.36 mL/g, ultrasonic time of 30 min, ultrasonic temperature of 45 °C, alcohol precipitation time of 6 h. The predicted polysaccharide yield under this condition was 20.54%. For ease of controlling the experimental conditions and convenience of operation, the parameters were adjusted to liquid-to-solid ratio of 40 mL/g, ultrasonic time of 30 min, ultrasonic temperature of 45 °C and alcohol precipitation time of 6 h. The average polysaccharide extraction yield was 19.93% after three parallel experiments under this optimal extraction process, confirming that the prediction model is effective and reliable.

### 2.5. Fourier−Transform Infrared (FT-IR) Spectroscopy

**Figure 4:**
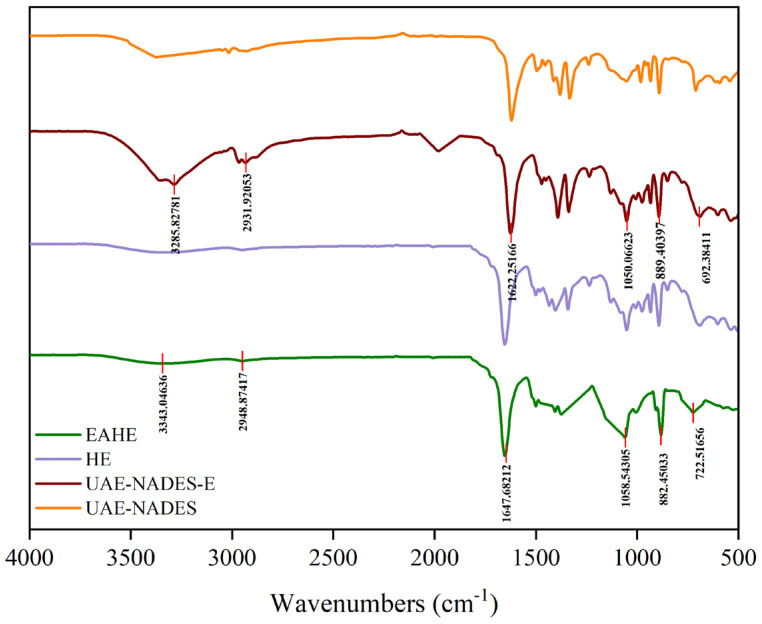
The FT−IR spectrum of polysaccharides from PDR by different extracting methods.

As shown in Figure 4, the FT-IR spectra of the polysaccharides obtained through four methods are similar, indicating the similar structure. There is a broad absorption peak in the scope at 3280–3350 cm^−1^, which is the stretching vibration peak of the O-H functional group in polysaccharides. The small absorption peak at 2931.92 cm^−1^ is related to the C-H stretching vibration of carbohydrates in carbohydrate groups, which is a characteristic peak of sugars. The spectral band at 1622.25–1647.68 cm^−1^ is the stretching vibration and asymmetric stretching vibration of the carbonyl C=O group in polysaccharides; the vibration around 1410 cm^−1^ is the variable angle bending vibration of the C-H bond. The absorption peak near 1050 cm^−1^ is the absorption peak of the C-O-C group in polysaccharides, the peak near 880 cm^−1^ indicates that the sugar is mainly composed of pyranose sugar rings. Critically, the absorption peaks near 1050 cm^−1^ and 880 cm^−1^ further provided the evidence for the α-configuration and α-1,6-linkage [28].

### 2.6. Monosaccharide Compositions of Polysaccharides

The composition of polysaccharides extracted via the above four methods was detected by ion chromatography and a total of 13 common monosaccharides were used as reference substances (Figures S1–S5, Supplementary Materials). Eight monosaccharides were detected in the extracted polysaccharides, namely Ara (Arabinose), Rha (Rhamnose), Gal (Galactose), Glc (Glucose), Man (Mannose), Xyl (Xylose), Gal-Ua (Galacturonic acid) and Glc-Ua (Glucuronic Acid), the molar ratio of these monosaccharides were a little different across the extraction methods (Table 3). The major components of PDR polysaccharides were Ara, Gal and Glc, the total proportion of these three monosaccharides were 86.24%, 88.6%, 89.19% and 91.6% extracted by HE, EAHE, UAE-NADES and UAE-NADES-E, respectively. The results suggest that the enzymolysis and NADESs improved the extraction efficiency and the purity of polysaccharides [29].

### 2.7. Molecular Weight of Polysaccharides

The molecular weight (MW), including the number average molecular weight (Mn), the average molecular weight of z (Mz), the weight average molecular weight (Mw) and the peak molecular weight (Mp, refers to the molecular weight of the component fragment with the highest content) was determined by gel chromatography coupled with differential multi-angle laser light scattering system. As shown in Figures S6–S9 (Supplementary Materials), the molecular mass (black line) was obtained by fitting the multi-angle laser light scattering signal (i.e., LS, unit: V) and differential signal (i.e., RI, unit: RIU).

Table 4 reveals that the MW of the PDR polysaccharides varied depending on the extraction methods. Compared to the HE, the MW of EAHE, UAE-NADES and UAE-NADES-E produced lower MW polysaccharides. First, the cavitation and mechanical effects of sonication might destroy the spatial structure of PDR polysaccharides and result in the low MW. Secondly, the combination of ultrasound, NADESs and the enzyme could further destroy the spatial structure, the glycosidic bonds and the branched chain structure, and thus the much lower MW of PDR polysaccharides was obtained. The polydispersity index (Mw/Mn) is the width index of the molecular weight distribution. In this study, the Mw/Mn varied significantly across the extraction methods. The Mw/Mn of PDR polysaccharides extracted by HE and EAHE is 1.712 and 1.899 (<2), respectively, which indicate that the polysaccharides have good uniformity. While the Mw/Mn of PDR polysaccharides extracted by UAE-NADES and UAE-NADES-E were 2.409 and 3.110, respectively, indicating a significant difference in the molecular weight.

### 2.8. Surface Morphology of Polysaccharides Observed by SEM

**Figure 5:**
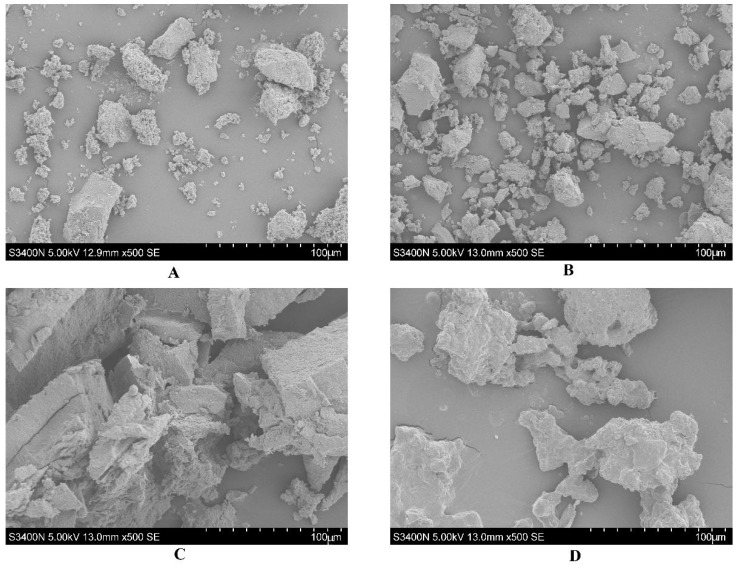
The microscopic structure of the PDR polysaccharides by HE method (**A**), EAHE method (**B**), UAE-NADES method (**C**) and UAE-NADES-E method (**D**).

The surface morphology of polysaccharides obtained by different extraction methods at the same magnification (100 μm) are shown in Figure 5, revealing method-dependent characteristics. The HE yielded irregular block-like polysaccharide particles, relatively dispersed distribution, smooth particle surface and a compact structure (Figure 5A). The EAHE significantly reduced the particle size of polysaccharides, increased the number of fine particles, reduced agglomeration, and increased the degree of structural looseness (Figure 5B). The UAE-NADES generated layered or flaky broken structures of polysaccharide particles, with obvious internal pores and intensified structural damage. The surface was rough and had a rich sense of layering. The UAE-NADES-E resulted in a more irregular morphology of polysaccharide particles, with some particles exhibiting pore structures (Figure 5D), indicating the most significant damage and highly fragmented characteristics overall. The above results indicated that hot water extraction relied only on temperature diffusion, resulting in limited disruption of polysaccharide structure and retention of relatively intact block-like morphology. Enzymatic hydrolysis disrupted the intermolecular forces of the polysaccharides, leading to particle fragmentation, refinement and structural looseness. The polysaccharide aggregation structure was further disrupted by the cavitation effect of ultrasound and the solubility of NADESs; the layered and porous structures were formed, and the specific surface area was increased. The UAE-NADES-E combined ultrasonic physical fragmentation, solvent dissolution and enzymatic chemical reactions to synergistically destroy the surface structure of polysaccharides, resulting in the highest degree of fragmentation and porosity, which may be more conducive to the exposure and subsequent utilization of polysaccharide active sites.

### 2.9. The Cycle Extraction of Polysaccharides by NADESs

The NADES recycling was carried out by following the method [30] of Li et al., the details are provided in the Supplementary Materials. As Figure S10 (Supplementary Materials) demonstrates, the polysaccharide extraction yield progressively declined from 19.85% to 15.14% over five cycles., while the NADES-6 recovery rate decreased from 97.87% to 88.13% over five cycles. These results confirmed that NADES-6 has excellent reproducible extraction efficiency and recyclability. The decrease in the continuous cycle extraction efficiency may be due to the repeated use of hydrogen bond breakage in NADES-6, resulting in a decrease in performance.

### 2.10. Antioxidant Activity Results

Plant polysaccharides are recognized as natural active ingredients demonstrating various biological activities, including anti-cancer, antiviral and antioxidant properties, making them an important source for developing new natural antioxidants. In this work, the antioxidant activity of the polysaccharides extracted from the above four methods have been investigated by the DPPH, ABTS and hydroxyl radical scavenging activities with VC as the control.

**Figure 6:**
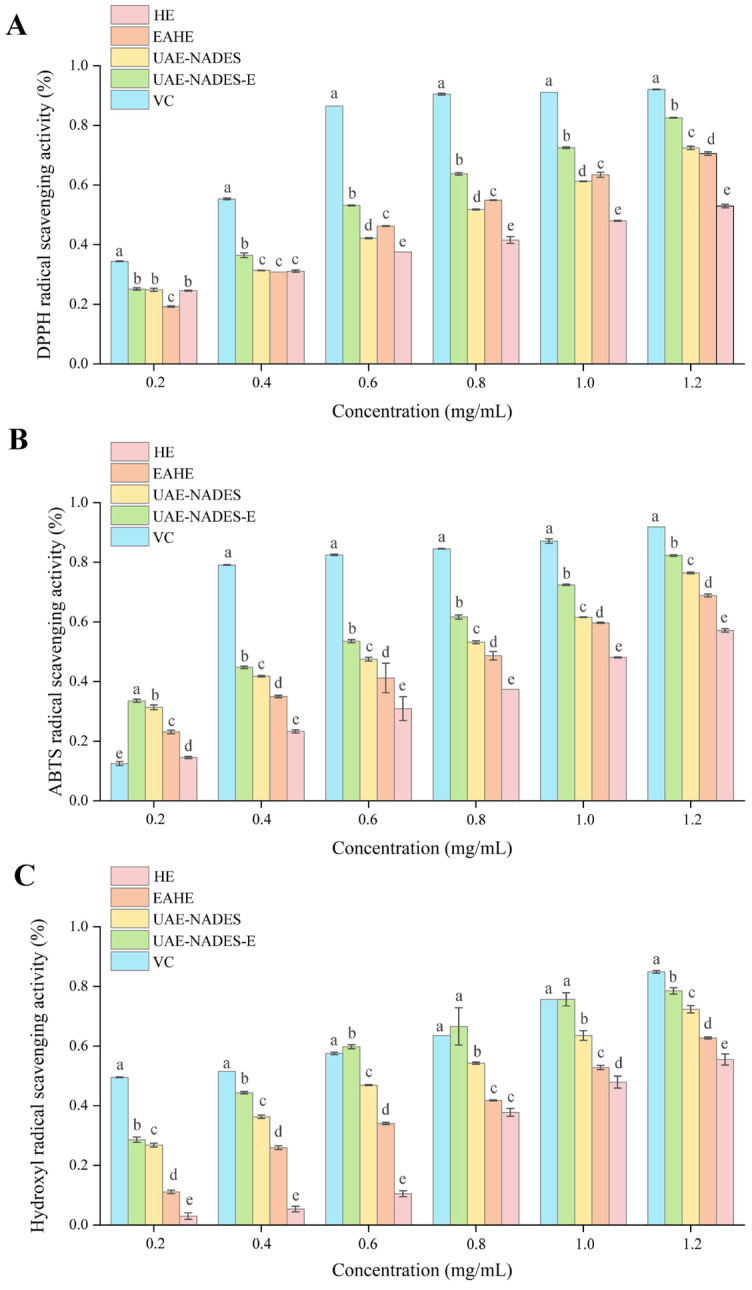
Antioxidant activity of polysaccharides on scavenging activity to DPPH radicals (**A**), scavenging activity to ABTS radicals (**B**) and scavenging activity to hydroxyl radicals (**C**).

Figure 6 illustrates that as the quality concentration of the PDR polysaccharides extract increases, the clearance ability of the different solution extracts shows an increasing trend. Notably, polysaccharides extracted using UAE-NADES-E method had a higher antioxidant capacity compared to other extraction methods. Take DPPH clearance ability as an example, when the concentration of the VC control solution reached 0.50 mg/mL, the removal rate of DPPH free radicals tends to stabilize. The NADES extract had a higher removal rate of DPPH with increasing concentration, reaching 82.08% at 1.2 mg/mL. Conversely, the DPPH clearance capacity of the polysaccharides obtained by hot water extraction method is only 57.62%, at the specified concentration. The scavenging activities of ABTS and hydroxyl radical showed a similar trend. 

A negative control test was conducted using blank NADES-6 without sample addition. The results (Figure S11, Supplementary Materials) showed that the DPPH, ABTS and hydroxyl radical scavenging rates of NADES-6 were all below 5%, significantly lower than those of extracted polysaccharides. This confirmed that the residual NADES component in polysaccharides contributes very little to the antioxidant activity.

The PDR polysaccharides extracted by the UAE-NADES-E method exhibited significantly enhanced clearance ability for free radicals than those obtained by hot water extraction. The in vitro antioxidant capacity of polysaccharides is significantly influenced by the physicochemical properties, including molecular weight, monosaccharide compositions and types. Generally, low molecular weight polysaccharides are more likely to bind with free radicals due to their more porous structure, larger surface area and more reducing hydroxyl end groups [31,32,33]. In this work, the polysaccharides extracted using UAE-NADES-E method have a lower molecular weight and higher purity, hence have higher antioxidant capacity.

## 3. Materials and Methods

### 3.1. Materials and Regents

The plant materials were collected from Dejiang county, Guizhou province, China. The voucher specimens, (NO. GAUAB-PD-20231010), were identified as umbelliferous plant *Peucedanum decurvivum* (Miq.) Maxim by Professor Yuan Chen (Gansu Agricultural University) and deposited. The plant samples were dried in an oven at 40 °C for 24 h and then crushed using a small high-speed grinder. The crude PDR powder was prepared by sieving through No. 4 sieve (40 mesh, 0.45 mm), and stored in a refrigerator at 4 °C.

The chemical regents used in this work including methanol, anhydrous ethanol, choline chloride, betaine, lactic acid, critic acid, glucose, glycol, 1, 2-propylene glycol, 1,3-butanediol, 1,4-butanediol, n-butyl alcohol, sulfuric acid, 1,1-diphenyl-2-picrylhydrazyl (DPPH), diammonium 2,2′-azino-bis (3-ethylbenzothiazoline-6-sulfonate) (ABTS), 30 % hydrogen peroxide, cellulase (lot: CC31152810), phenol and CHCl_3_ were purchased from commercial sources with analytical purity grade.

### 3.2. Preparation of NADESs

In this work, the NADESs were prepared by a simple heating–stirring experiment. The hydrogen bond donors and hydrogen bond acceptors were mixed according to certain molar ratios and placed in a 250 mL conical bottle, which were reacted at 80 °C in a water bath and stirred continuously until a stable and uniform transparent liquid was formed [34,35,36,37,38]. Six different NADES systems with choline chloride and betaine as hydrogen bond acceptors were prepared in this study (Table 5).

### 3.3. Determination of Polysaccharide Extraction Yields

The extraction yields of polysaccharides was determined using phenol–sulfuric acid method [39] by UV spectrophotometer (T6) at 490 nm. The standard curve was drawn and the equation was Y = 0.0067X + 0.1844, R^2^ = 0.9991, indicating a good linear relationship. The detailed experiments are provided in the Supplementary Materials.

### 3.4. Extraction of Polysaccharides from PDR

#### 3.4.1. Pretreatment of PDR Powder

An appropriate amount of anhydrous ethanol was added to the PDR powder and the mixture was heated reflux for 1.5 h (three times). The pre-treated powder was obtained by drying the residue under vacuum at 40 °C.

#### 3.4.2. Extraction of Polysaccharides

The following extracting experiments were repeated three times.

Extraction Method 1: Hot water extraction (HE) [40]

The pre-treated PDR medicinal powder (5.00 g) was added into distilled water at a liquid-to-material ratio of 20:1 mL/g and extracted at 90 °C for 2 h. The mixture was then filtered while hot, and the filter residue was retained and extracted three times. The filtrate was combined and concentrated, an appropriate amount of anhydrous ethanol added, which was let stand at 4 °C for 12 h. After centrifuging, the polysaccharides were obtained through the deproteinization of the precipitate.

Extraction Method 2: Enzyme-assisted hot water extraction (EAHE) [40]

The pre-treated PDR medicinal powder (5.00 g) was mixed with distilled water at a liquid-to-material ratio of 20:1 mL/g, 0.01 g cellulase was added to the system and was soaked in water at 90 °C for 2 h. Then it was filtered while hot, and the filter residue was retained and extracted three times. The filtrate was combined and concentrated, an appropriate amount of anhydrous ethanol added, which was let stand at 4 °C for 12 h. After centrifuging, the polysaccharides were obtained through the deproteinization of the precipitate.

Extraction Method 3: Ultrasonic-assisted extraction with NADESs (UAE-NADES) [25]

The pre-treated PDR powder (5.00 g) was mixed with NADES aqueous solution (100 mL). The ultrasonic-assisted extraction with NADESs was carried out in an ultrasonic bath with a power of 300 W, 50 Hz, time of 40 min and temperature of 45 °C. The filtrate was concentrated to one-third of its original volume using a rotary evaporator under reduced pressure. Then the concentrate was diluted with anhydrous ethanol at 1:4 (*v*/*v*) ratio, followed by storage in a refrigerator at 4 °C for 12 h. The polysaccharides were obtained after the deproteinization of the precipitate.

Extraction Method 4: Ultrasonic-assisted extraction with NADESs and enzyme pretreatment (UAE-NADES-E) [25]

The pre-treated PDR powder (5.00 g) was mixed with NADES aqueous solution (100 mL), and cellulase (0.01 g) was added. The ultrasonic-assisted extraction was carried out in an ultrasonic bath with a power of 300 W, 50 Hz, time of 40 min and temperature of 45 °C. The filtrate was concentrated to one-third of its original volume using a rotary evaporator under reduced pressure. Then the concentrate was diluted with anhydrous ethanol at 1:4 (*v*/*v*) ratio, placed in a refrigerator at 4 °C for 12 h. The polysaccharides were obtained after the deproteinization of the precipitate.

#### 3.4.3. Deproteinization

The deproteinization of crude polysaccharides was performed by the Sevage method [40]. Sevage reagent was prepared with trichloromethane and n-butanol at a certain volume ratio and then mixed with crude polysaccharide solution at 1:5 (*v*/*v*) ratio. The system was left for 20 min, followed by centrifugation at 8000 r/min for 15 min. The above operations were repeated five times. When no denatured protein at the junction was observed between the supernatant and the organic layer, the supernatant was collected and dried under vacuum to obtain the PDR polysaccharides.

### 3.5. Optimization of Extraction Process of Polysaccharides from PDR

#### 3.5.1. Single Factor Test

The effects of the liquid/solid ratio (10, 20, 30, 40, 50 mL/g), ultrasonic time (20, 30, 40, 50, 60 min), ultrasonic temperature (25, 35, 45, 55, 65 °C) and ethanol precipitation time (2, 6, 12, 18, 24 h) on the extraction yields of PDR polysaccharides were investigated in triplicate.

#### 3.5.2. BBD-RSM Test

A Box–Behnken design (BBD)–response surface methodology (RSM) optimizing experiment [41] was designed based on the results of single factor test. A total of 29 experiments based on 4 factors and 3 levels were carried out in triplicate.

### 3.6. Determination of Antioxidant Activity of Polysaccharides

The antioxidant activity of polysaccharides was determined by using the DPPH, ABTS radical and hydroxyl radical scavenging assays, with Vitamin C (VC) as a positive control, which were measured by the UV-Vis Spectrophotometer. The experiments in this work were carried out according to the previous reports with sight modifications [41,42,43].

### 3.7. Characterization of Polysaccharides

The IR spectrum of polysaccharides from PDR were carried out by a PerkinElmer FT-IR Spectrum 3 instrument (PerkinElmer Corporation, Waltham, MA, USA). The FT-IR spectra were collected in the mid-infrared region of 4000–500 cm^−1^. The homogeneity and molecular weight of polysaccharides from PDR were determined by Size Exclusion Chromatography–Multi-Angle Light Scattering–Refractive Index (SEC-MALLS-RI) with DAWN HELEOS-II laser photometer (Wyatt Technology Co., Santa Barbara, CA, USA). The monosaccharide composition and content was determined by high-performance anion-exchange chromatography (HPAEC) (ICS 5000+, Thermo Fisher Scientific, Waltham, MA, USA). The microscopic features and differences of the PDR polysaccharides extracted by four methods was investigated by scanning electron microscopy (SEM) (JEOL S-3400SN, HITACHI, Chiyoda, Tokyo, Japan) analysis.

### 3.8. Statistical Analysis

The software Design-Expert 13 was used for the Box–Behnken test design and analysis of variance. Origin 2021 was used to draw the experimental data to images. The experiments in this work were conducted using technical replicates, with each experiment repeated three times.

## 4. Conclusions

In this study, the optimal NADES system for extracting polysaccharides from PDR was screened out and the extraction process was optimized by using response surface methodology. The results showed that the effect of extraction conditions on polysaccharide extraction yields followed this order: liquid to material ratio > ultrasonic temperature > alcohol precipitation time > ultrasonic time. Compared to the traditional hot water reflux method, the UAE-NADES-E has the advantage of a higher extraction yield and shorter extraction time. FT-IR spectroscopy suggested that the polysaccharides obtained from the four methods possessed the similar structure features. The extraction methods did not change the monosaccharide composition of the polysaccharides, but the total proportion of the major monosaccharides Ara, Gal and Glc was notably increased by the UAE-NADES-E method. However, the mechanism regarding the effects of NADESs on the structural properties and biological effects of PDR polysaccharides has not been revealed. The biological activity and activation mechanism of PDR polysaccharides extracted by NADESs will be studied for future application in the food and biomedical fields.

## Figures and Tables

**Table 1:** The experimental factors and levels by BBD.

Factors	Levels		
	−1	0	1
A (liquid-to-material ratio, mL/g)	20	30	40
B (ultrasonic time, min)	30	40	50
C (ultrasonic, temperature, ℃)	25	35	45
D (alcohol precipitation time, h)	6	12	18

**Table 2:** Response surface experimental design and the extraction yields.

NO.	A	B	C	D	Y (Yields, %)
1	1	0	0	−1	6.46
2	1	0	0	1	11.87
3	0	0	0	0	7.34
4	0	0	−1	−1	12.22
5	1	−1	0	0	13.77
6	1	0	−1	0	6.52
7	0	0	0	0	7.21
8	−1	0	−1	0	3.22
9	−1	1	0	0	8.44
10	−1	0	−1	0	1.86
11	−1	0	0	1	1.70
12	0	1	0	1	12.74
13	0	−1	−1	0	14.53
14	0	0	−1	−1	2.85
15	−1	0	0	−1	5.52
16	0	−1	0	−1	12.34
17	0	0	−1	1	8.92
18	0	1	0	−1	10.70
19	0	0	−1	1	5.62
20	0	1	−1	0	8.12
21	1	0	−1	0	8.29
22	0	−1	0	1	10.85
23	1	1	0	0	8.29
24	0	0	0	0	7.77
25	−1	−1	0	0	3.68
26	0	0	0	0	7.47
27	0	1	−1	0	12.28
28	0	−1	−1	0	4.59
29	0	0	0	0	7.03

**Table 3:** The monosaccharide compositions of polysaccharides obtained by various methods.

Extraction Methods	Ara	Rha	Gal	Glc	Xyl	Man	Gal-UA	Glc-UA
HE	13.27%	2.20%	19.65%	53.32%	0.92%	1.32%	7.97%	1.35%
EAHE	12.06%	2.00%	18.65%	57.89%	0.84%	1.37%	5.97%	1.22%
UAE-NADES	27.89%	2.58%	40.56%	20.74%	1.19%	1.85%	2.32%	2.86%
UAE-NADES-E	17.77%	2.15%	31.58%	42.25%	0.95%	1.59%	2.14%	1.56%

**Table 4:** Molecular weight of polysaccharides obtained by various methods.

Extraction Methods	Mn (kDa)	Mp (kDa)	Mw (kDa)	Mz (kDa)	Polydispersity (Mw/Mn)
HE	81.467	108.201	139.499	429.715	1.712
EAHE	44.935	17.970	85.332	137.667	1.899
UAE-NADES	38.468	52.769	92.686	295.408	2.409
UAE-NADES-E	16.474	8.801	51.233	230.688	3.110

**Table 5:** The NADES systems prepared in this work.

NO.	Hydrogen Bond Acceptors (HBA)	Hydrogen Bond Donors (HBD)	HBA/HBD Ratio	Water Content
1	Choline chloride	citric acid	1:3	30%
2	Choline chloride	1, 2-propylene glycol	1:3	30%
3	Choline chloride	glycol	1:3	30%
4	Betaine	1,4-butanediol	1:3	30%
5	Betaine	lactic acid	1:3	30%
6	Betaine	1,3-butanediol	1:3	30%

## Data Availability

The original contributions presented in this study are included in the article/Supplementary Materials. Further inquiries can be directed to the corresponding author.

## Supplementary Materials

The following supporting information can be downloaded at: https://www.mdpi.com/article/10.3390/plants14142188/s1, 1. Calculation of polysaccharides extraction yields; 2. Methodological validation; 3. NADES recycling experiment; Figure S1: The Ion Chromatogram of mixed standard curve; Figure S2: The Ion Chromatogram of polysaccharides extracted by HE method; Figure S3: The Ion Chromatogram of polysaccharides extracted by EAHE method; Figure S4: The Ion Chromatogram of polysaccharides extracted by UAE-NADES method; Figure S5: The Ion Chromatogram of polysaccharides extracted by UAE-NADES-E method; Figure S6: Absolute molecular weight analysis diagram of polysaccharides extracted by HE method; Figure S7: Absolute molecular weight analysis diagram of polysaccharides extracted by EAHE method; Figure S8: Absolute molecular weight analysis diagram of polysaccharides extracted by UAE-NADES method; Figure S9: Absolute molecular weight analysis diagram of polysaccharides extracted by UAE-NADES-E method; Figure S10: Effect of repeated use of NADES on the yield of polysaccharides; Figure S11: Antioxidant activity of NADES-6 on scavenging activity to DPPH radicals (A), scavenging activity to ABTS radicals (B) and scavenging activity to hydroxyl radicals (C); Table S1: Response surface data analysis of variance.
