# Supplementary material for: An Efficient Extraction, Characterization and Antioxidant Study of Polysaccharides from Peucedani Decursivi Radix
Source: Plants (Basel). 2025 Jul 15;14(14):2188. doi: 10.3390/plants14142188 (PMC12298027; doi:10.3390/plants14142188)
Supplement: Supplementary file 1 [file plants-14-02188-s001.zip › plants-3696693-supplementary.pdf]

Supplementary material

An efficient extraction, characterization and antioxidant study of polysaccharides from *Peucedani Decursivi* Radix

Qian Li, Zeyu Li, Chaogui Hu, Chenyue Wang, Feng Yang, Xiaoqin Ding

State Key Laboratory of Aridland Crop Science, College of Agronomy, Gansu Agricultural University, Lanzhou 730070, China

Correspondence: Qian Li, Email: liqian1984@gsau.edu.cn

1. Calculation of polysaccharides extraction yields

First, 1 mg/mL glucose standard solution was prepared and stored it in a refrigerator at 4 ℃ for later use. Then, appropriate amount of standard solution was taken and diluted at different concentrations. After standing for a period of time, 1 mL of 5% phenol solution and 4 mL of concentrated sulfuric acid were added, shaken, placed for 15 min, then removed in a boiling water bath for 15 min, and cooled to room temperature. The absorbance was measured at 490 nm by ultraviolet spectrophotometer. The standard curve is drawn with the mass concentration of glucose as the horizontal coordinate and absorbance as the vertical coordinate. The standard curve equation is Y=0.0067X+0.1844, R^2=0.9991, indicating a good linear relationship in the range of 10 -50 μg/mL.

The extracted polysaccharides were weighed and the polysaccharides solution was fixed to 10 mL with distilled water. The absorbance of the solution was determined at 490 nm. The following formula was used to calculate the polysaccharides yield (Y) combined with the standard curve equation.

$$Y = \frac{c \times v}{m} \times 100\%$$

In the formula, c is the mass concentration of polysaccharides in solution mg/mL, v is the polysaccharides solution volume, m is the mass of polysaccharides g.

2. Methodological Validation

2.1 Precision Test

The prepared standard solution was measured with repeated six times according to the above operation steps, and the absorbance values was recorded. The RSD value was calculated to be 0.12%, indicating that the instrument has good precision.

2.2 Stability Test

The absorbance of the prepared sample solution was measured every 10 min within 1 h, with the repetition of six times. The RSD value was calculated to be 2.34%, indicating that the sample solution prepared in the experiment is stable within 1 h.

2.3 Repeatability Test

The five samples of PDR powder were prepared as the sample solutions, and their absorbance values were successively measured. The RSD value of the five sample solutions was calculated to be 1.54%, indicating that the method has good reproducibility.

3. **NADES recycling experiment**

We followed the method of Li et al. and precipitated polysaccharides with ethanol under optimal extraction conditions, and collected the resulting solution. Rinse the storage container with a small amount of anhydrous ethanol. After combining the rinsing solution with the collected solution, use a rotary evaporator to remove the ethanol. Perform five cycles in total to calculate the recovery rate of NADES-6 and polysaccharide extraction rate. The recovery rate of NADES-6 is determined as shown in the formula.

$$\text{NADES recovery (\%)} = \frac{Recovery\ volume\ V1}{Recovery\ volume\ V2} \times 100\%$$

Where V1 represents the volume (mL) recovered after rotary evaporation, and V2 represents the volume (mL) added during NADES-6 polysaccharide extraction.

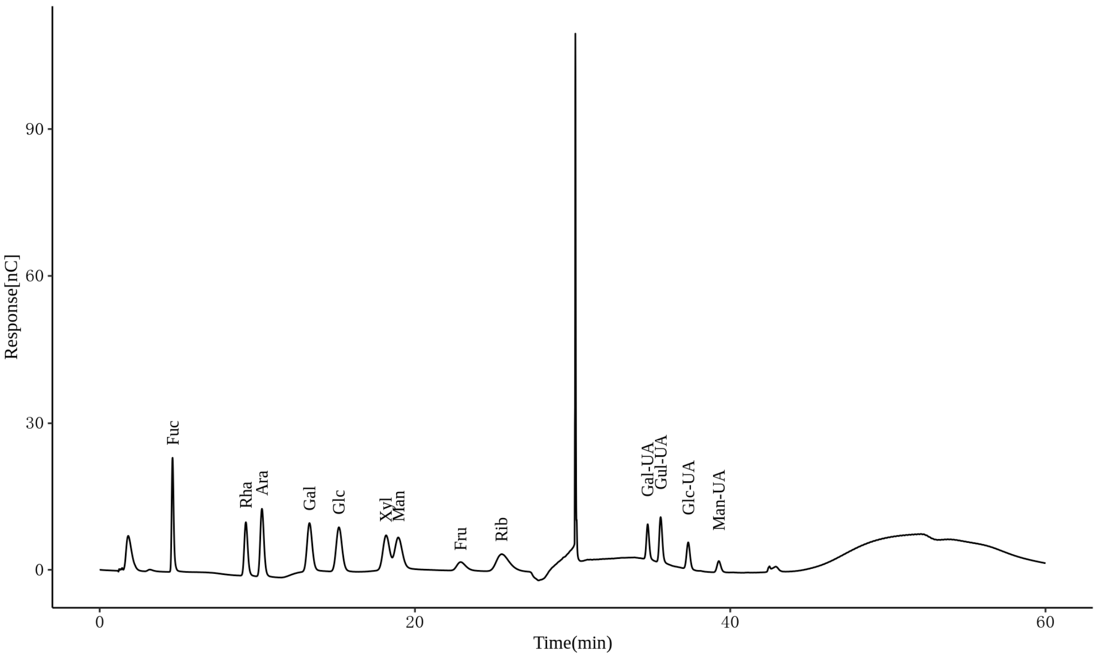

Figure S1 The Ion Chromatogram of mixed standard curve

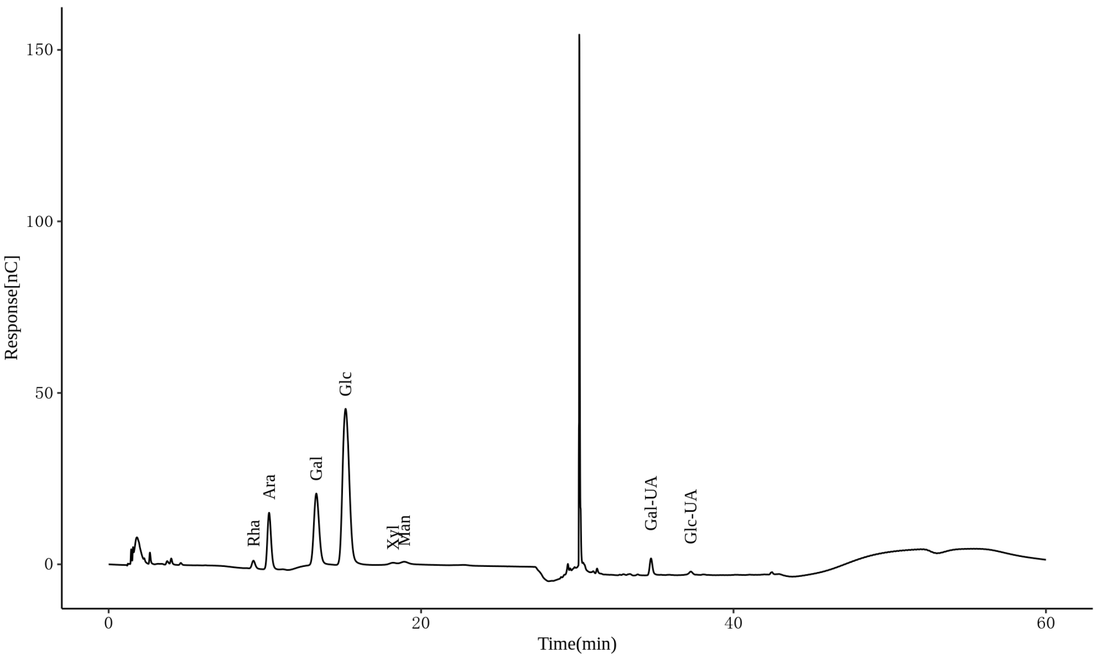

Figure S2 The Ion Chromatogram of polysaccharides extracted by HE method

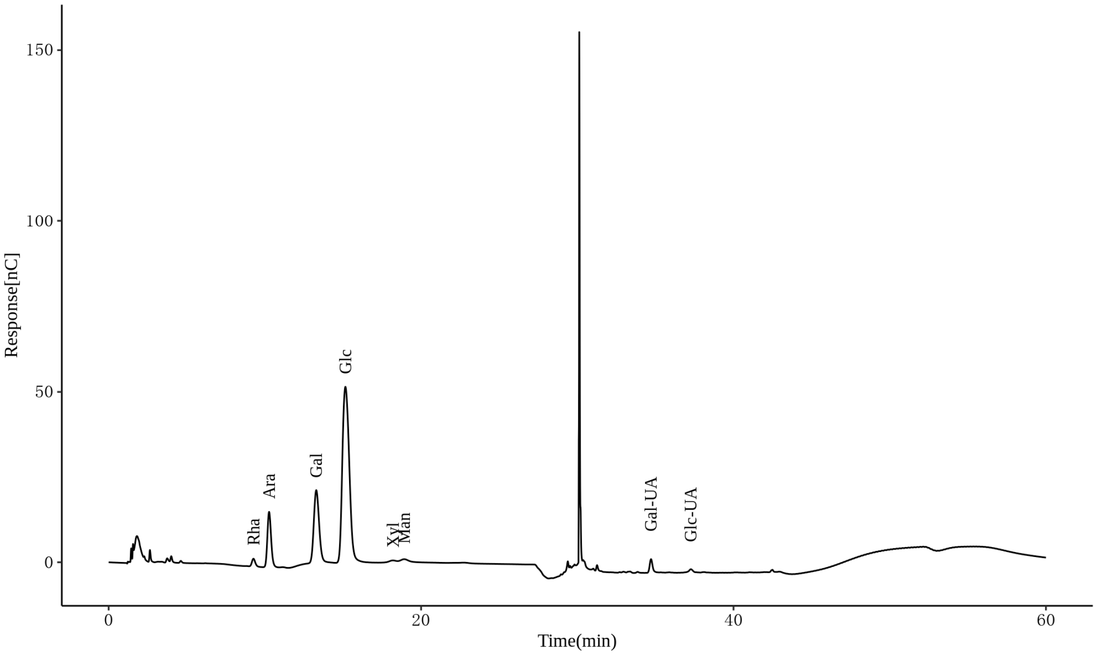

Figure S3 The Ion Chromatogram of polysaccharides extracted by EAHE method

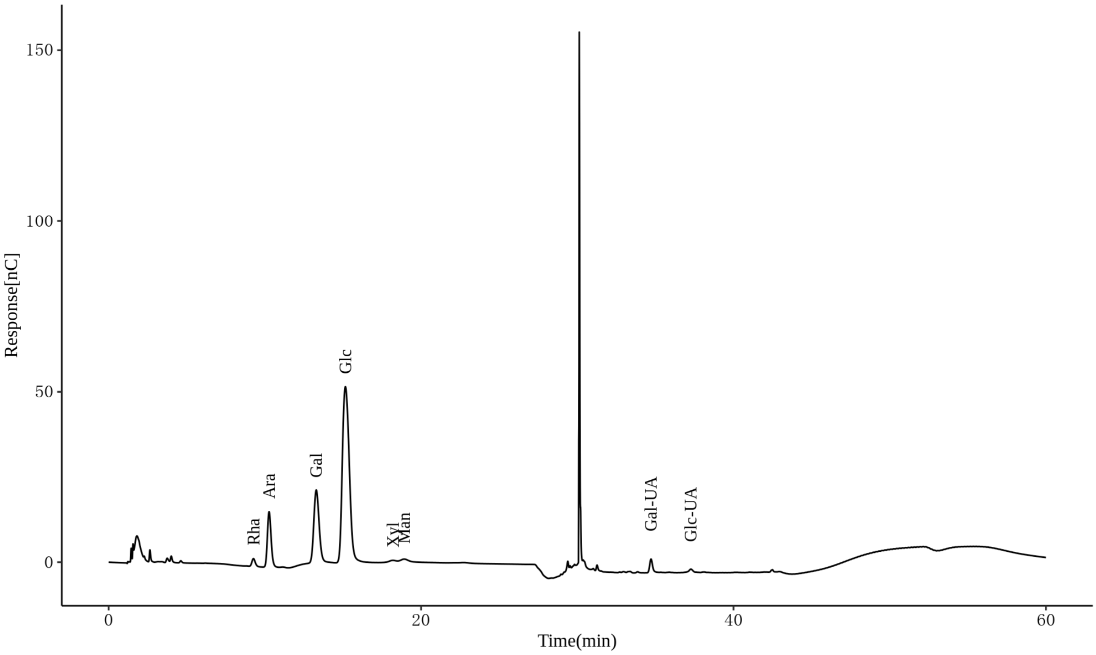

Figure S4 The Ion Chromatogram of polysaccharides extracted by UAE-NADES method

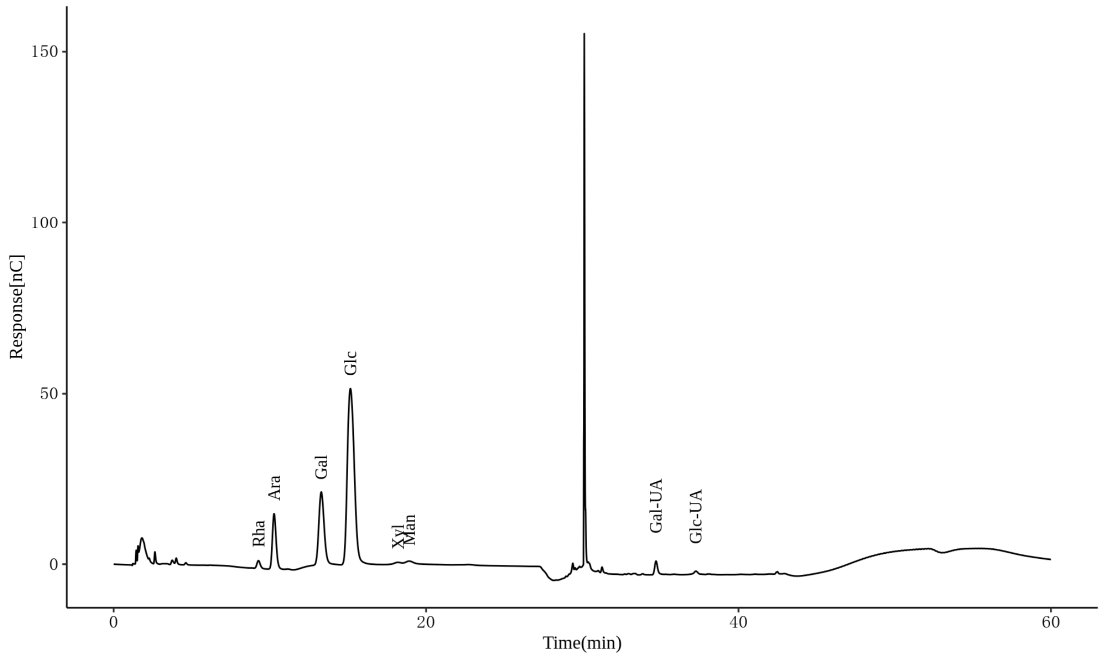

Figure S5 The Ion Chromatogram of polysaccharides extracted by UAE-NADES-E method

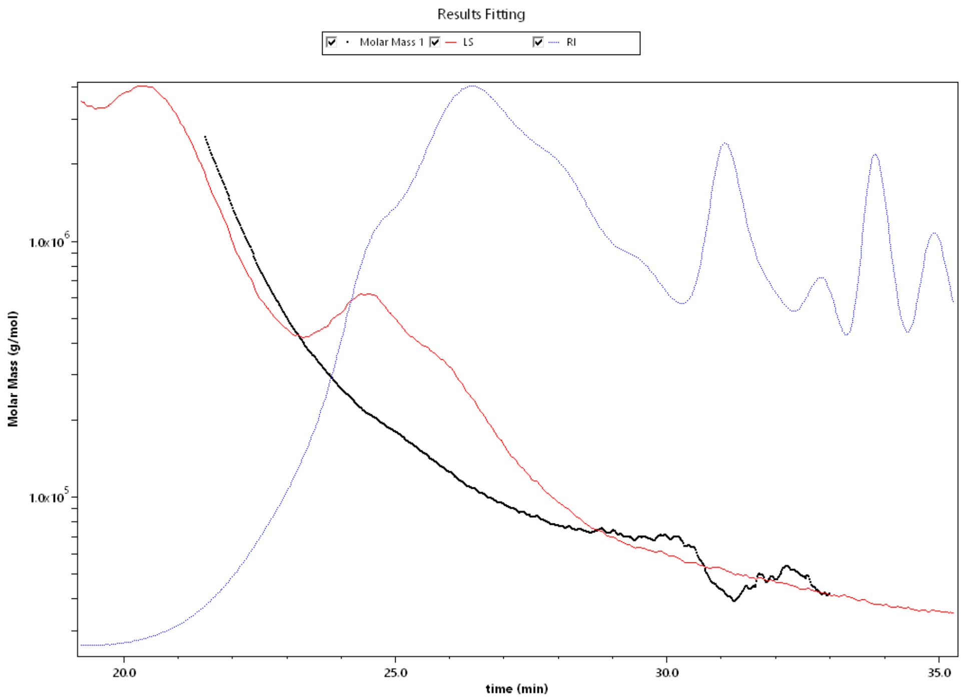

Figure S6 Absolute molecular weight analysis diagram of polysaccharides extracted by HE method

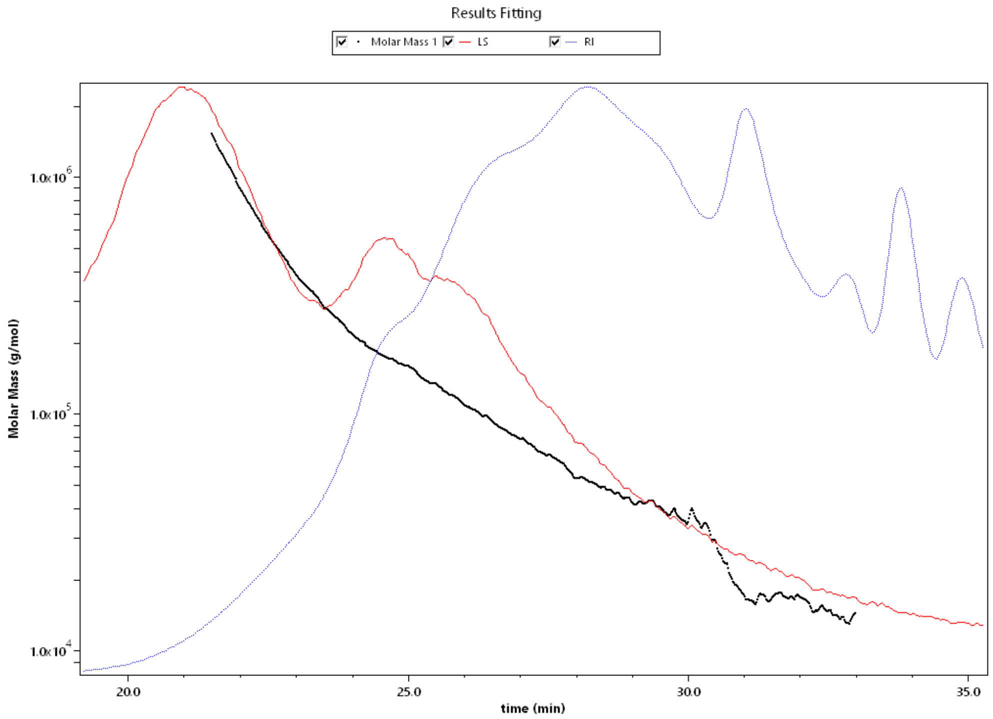

Figure S7 Absolute molecular weight analysis diagram of polysaccharides extracted by EAHE method

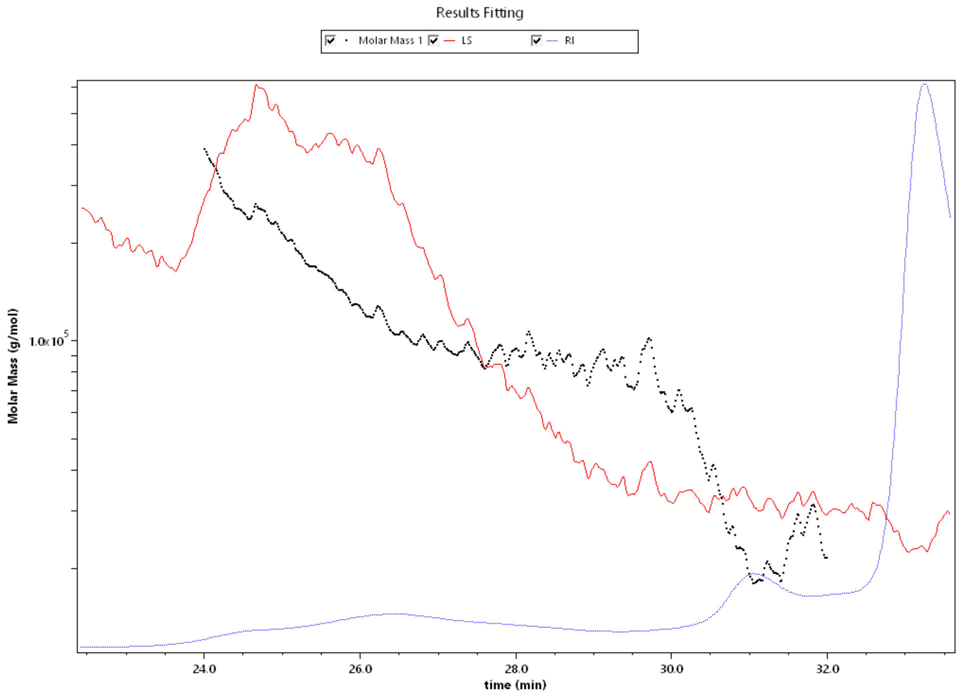

Figure S8 Absolute molecular weight analysis diagram of polysaccharides extracted by UAE-NADES method

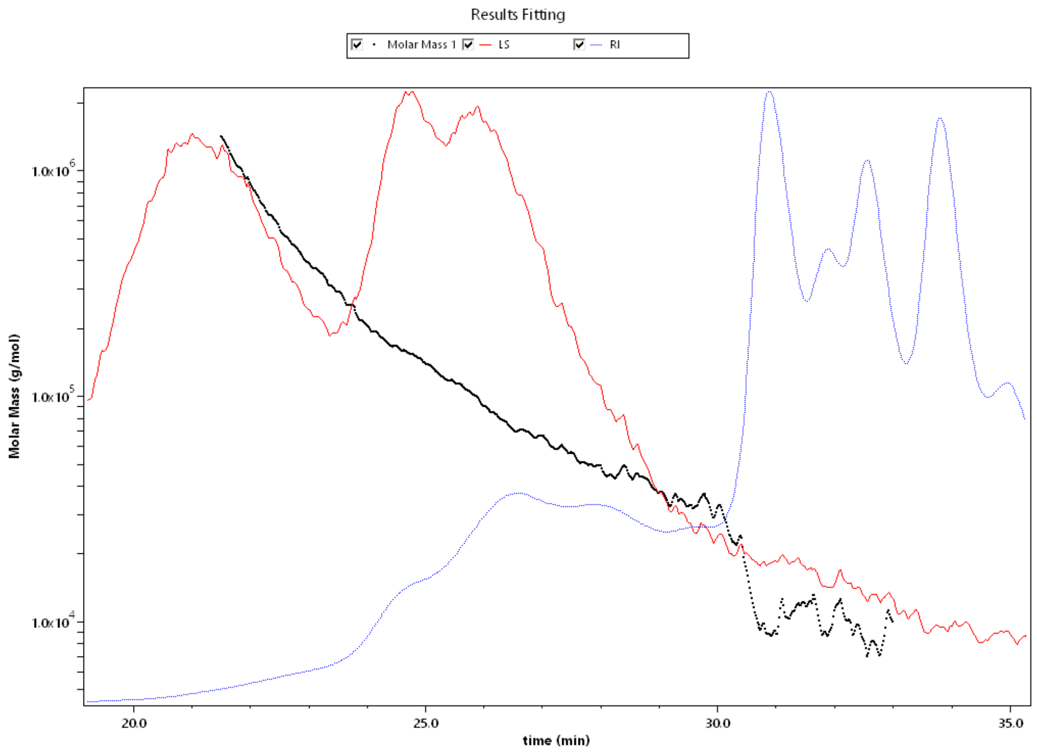

Figure S9 Absolute molecular weight analysis diagram of polysaccharides extracted by UAE-NADES-E method

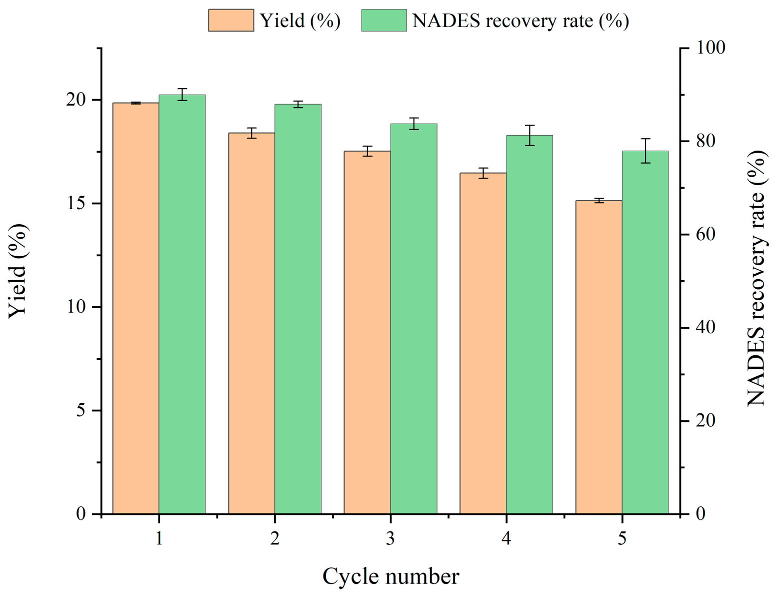

Figure S10 . Effect of repeated use of NADES on the yield of polysaccharides.

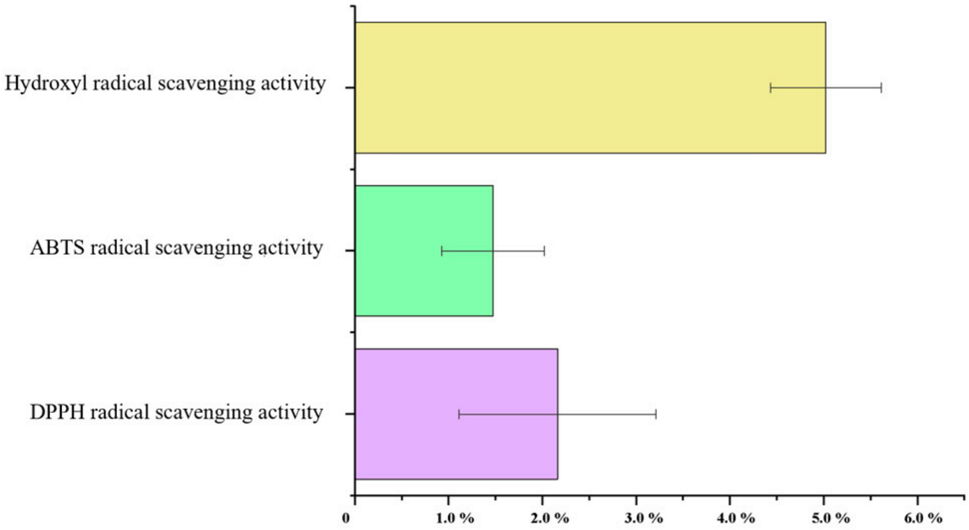

Figure S11 Antioxidant activity of NADES-6 on scavenging activity to DPPH radicals (A), scavenging activity to ABTS radicals (B) and scavenging activity to hydroxyl radicals (C).

Table S1 Response surface data analysis of variance

Source	Sum of Squares	df	Mean Square	F-value	p-value	Significance
model	357.35	14	25.52	104.11	< 0.0001	Significant
A	78.91	1	78.91	321.85	< 0.0001	
B	0.055	1	0.055	0.23	0.6421	
C	18.73	1	18.73	76.38	< 0.0001	
D	0.21	1	0.21	0.87	0.3674	
AB	26.19	1	26.19	106.82	< 0.0001	
AC	0.043	1	0.043	0.17	0.6834	
AD	21.30	1	21.30	86.86	< 0.0001	
BC	49.66	1	49.66	202.52	< 0.0001	
BD	3.10	1	3.10	12.66	0.0032	
CD	40.17	1	40.17	163.85	< 0.0001	
A^2	22.70	1	22.70	92.60	< 0.0001	
B^2	67.27	1	67.27	274.35	< 0.0001	
C^2	3.13	1	3.13	12.77	0.0031	

D^2	5.26	1	5.26	21.45	0.0004	
Residual	3.43	14	0.25			
Lack of Fit	3.12	10	0.31	3.94	0.0991	Not Significant
Pure Error	0.32	4	0.079			
Cor Total	360.78	28				
R^2	0.9905					
Adj R^2	0.9710					
